# Supplementary material for: Heme‐NO Dilates Arteries via Mobilization of NO Moieties From an Intracellular NO Store Within Vascular Smooth Muscle Cells
Source: Adv Sci (Weinh). 2026 May 8;13(43):e16857. doi: 10.1002/advs.202516857 (PMC13336038; doi:10.1002/advs.202516857)
Supplement: Supplementary file 1 — Supporting File: advs75612‐sup‐0001‐SuppMat.docx. [file ADVS-13-e16857-s001.docx]

**Supplemental Materials**

**Heme-NO Dilates Arteries via Mobilization of NO Moieties from an Intracellular NO Store within Vascular Smooth Muscle Cells**

Taiming Liu^1*^, Meijuan Zhang^1^, Lingchao Zhu^2^, Haiyan Ke^3^, Amancio de Souza^3^, Qian Li^4^, Daniel Castella^5^, Nicolai Lehnert^5^, Lubo Zhang^6^, Arlin B. Blood^1,6*^1) Department of Pediatrics, Division of Neonatology, Loma Linda University School of Medicine, Loma Linda, CA 92354, USA

2) Department of Chemistry, University of California, Riverside, USA

3) Metabolomics Core Facility, University of California, Riverside, USA

4) Department of Medicine, Gregory Fleming James Cystic Fibrosis Research Center, University of Alabama at Birmingham, USA

5) Department of Chemistry, University of Michigan, Ann Arbor, MI 48109-1055, USA

6) Lawrence D. Longo, MD Center for Perinatal Biology, Loma Linda University School of Medicine, Loma Linda, CA 92354, USA

^*^Correspondence to:

Taiming Liu. PhD, Department of Pediatrics, Division of Neonatology, Loma Linda University School of Medicine, Loma Linda, CA

Arlin Blood. PhD, Lawrence D. Longo, MD Center for Perinatal Biology, Loma Linda University School of Medicine, Loma Linda, CA.

Email: tliu@llu.edu; abblood@llu.edu

**Supplemental Methodologies**

**Wire myography.** Sheep mesenteric arteries were dissected from adult ewes, denuded of endothelium unless otherwise specified, and mounted in organ bath chambers (Radnoti Glass Instruments, Monrovia, CA) as previously described [^2^](https://paperpile.com/c/irc4iT/4AITK). Vessels were equilibrated for at least 30 min, tuned to a basal tension of 1 gram, constricted with 10 μM serotonin (5-HT) to achieve a comparable level of tension, and then subjected to accumulating additions of alb-heme-NO, PROLI NONOate (NO donor), or other drug. For some experiments, vessels were pre-exposed to three 15 min rounds of GSNO (5 μM) or UV light to deplete pre-existing NANOS before measurement of dilatory responses to alb-heme-NO. In some other experiments, 10 μM 1H-[1,2,4]oxadiazolo[4,3,-a] quinoxalin-1-one (ODQ), 10 μM nitrite, 200 μM 2-(4-Carboxyphenyl)-4,5-dihydro-4,4,5,5-tetramethyl-1H-imidazol-1-yloxy-3-oxide potassium salt (CPTIO), and/or 1000 U/ml Superoxide Dismutase 1 (SOD1) was added before tuning for basal tension. A dark beam SK68 LED flashlight was used to generate UV at 365 nm. To maximize the effects of UV, the organ bath was wrapped with foil film, while the UV light was shed downwards right from the top of the bath. Spontaneous tension changes in time matched control vessel rings were subtracted from individual experiments before calculation of relaxation.

**Artery homogenization.** Carotid arteries collected from experiments were extensively soaked and washed with ice-cold Hepes buffer for 45 min and then dried with Kimwipes before storage under -80 ^o^C (no snap frozen; AVOID liquid nitrogen). For homogenization, the arteries were thawed in Hepes buffer (1g in 5 ml; buffer contains 100 μM DTPA; no DTT or any other antioxidant), cut with scissors into small pieces, and then carefully homogenized on ice with a rotor-stator homogenizer (TissueRuptor, Qiagen Inc.; Hilden, Germany). The supernatant was collected after centrifugation at 6740 g for 30 s. Aliquots of the supernatant were either directly used for measurement or stored under -80 ^o^C (no snap frozen; AVOID liquid nitrogen) until analysis. Each aliquot was only allowed to be thawed once. Protein levels were measured by the Bradford assay.

**Chemiluminescence measurements.** The NOx levels were measured by a combo of five different assays with an ozone-based chemiluminescence NO analyzer (280i, Sievers, Boulder, CO) as previously described[^3,4^](https://paperpile.com/c/irc4iT/Ku32c+RlPUx). In the NiR+I_3_^-^ assay, the sample was first incubated with NiR (a mixture of nitrate reductase, FAD, and NADPH) at 37 ^o^C for 45 min and then subjected to analysis by the triiodide (I_3_^-^) assay. This assay measures nitrate in addition to targets of the I_3_^-^ assay. In the other four assays, the sample was injected into a purge vessel containing one of four reagents known to selectively convert various combinations of NO species into free NO gas to be carried into the NO analyzer by sparging with argon. These four assay reagents used were: 1) triiodide (I_3_^-^), 2) ascorbic acid + acetic acid (VitC+HAc), 3) potassium ferricyanide + ascorbic acid (FeCN+HAc), and 4) potassium ferricyanide + PBS (FeCN+PBS). The selectivity of the five assays are given in Table S1.

**GC-MS measurement.** The ^15^N NOx were measured under negative-ion chemical ionization mode by GC-MS (Agilent; 6890-5973) as previously described[^5,6^](https://paperpile.com/c/irc4iT/pXlkc+FWR7v). The GC-MS used helium as carrier gas and methane as reagent gas and was equipped with a OPTIMA-17 column (15 m × 0.25 mm × 0.25 µm) and a G2613A autosampler. For the sample preparation, 30 μl of biological fluid was spiked with 60 μl acetone, and 3 μl 2,3,4,5,6- pentafluorobenzyl bromide (PFB-Br; derivatization reagent). The mixture was incubated at 50 ^o^C for one hour. Then, 60 μl toluene was added to extract the derivative for measurement under selected ion monitoring mode (m/z=47 for nitrite+FeNO; m/z=63 for nitrate) using an external standard method. A standard curve of ^15^N-labeled nitrite and nitrate was made daily.

**Liquid Chromatography Mass Spectrometry (LC-MS^2^) measurements.** The measurements were performed on a G2-XS quadrupole time-of-flight mass spectrometer (Waters) coupled to an H-class UPLC system (Waters). Separations were carried out on a ACQUITY UPLC BEH C18 column (2.1 x 50 mm, 1.7 µM) (Waters). The mobile phases were (A) water with 0.1% formic acid and (B) acetonitrile with 0.1% formic acid. The flow rate was 350 µL/ min and the column was held at 40° C. The injection volume was 1 µL. The gradient was as follows: 0 min, 95% A; 0.5 min, 95% A; 2.5 min, 0% A; 4.5 min, 0% A; 5 min, 95% A; 7 min, 95% A. The MS was operated in Tof-MS mode. Source and desolvation temperatures were 150° C and 600° C, respectively. Desolvation gas was set to 600 L/hr and cone gas to 150 L/hr. Collision gas was set to 0.15 mL/min. All gases were nitrogen except the collision gas, which was argon. Capillary voltage was 2 kV in positive ion mode.

**Table S1.** Capability of different purge vessel reagents for the detection of different NO species.

| Reagent | NOx | | | | | |
| --- | --- | --- | --- | --- | --- | --- |
|  | nitrate | nitrite | SNOs | FeCN | | |
|  |  |  |  | BDNIC | MDNIC | heme-NO |
| NiR+I_3_^-^ | + | + | + | + | + | + |
| I_3_^-^ | - | + | + | + | + | + |
| VitC+HAc | - | + | - | - | - | + |
| FeCN+HAc | - | + | - | + | + | + |
| FeCN+PBS | - | - | - | + | + | + |

+: detectable; -: not detectable.


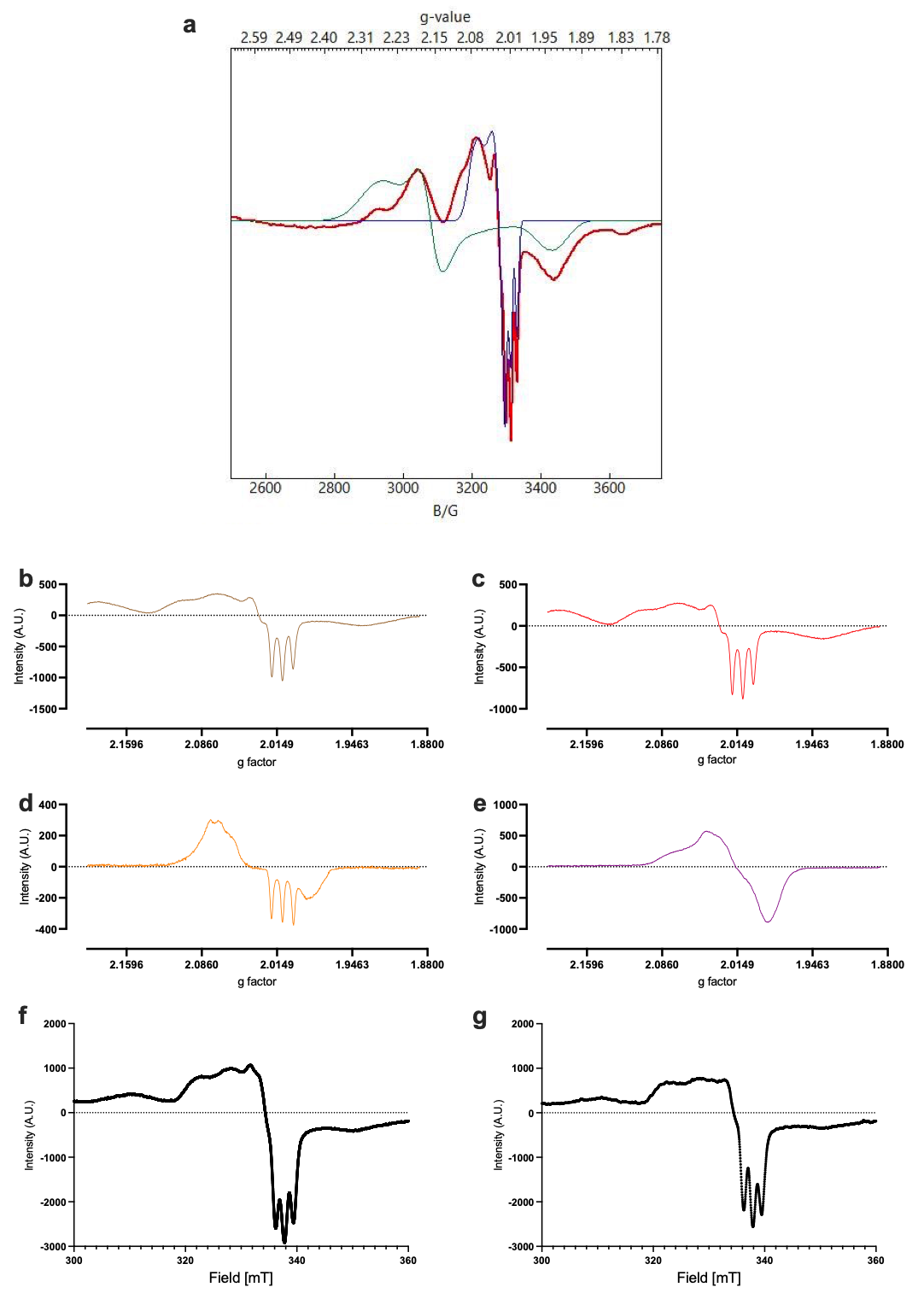


Figure S1. Comparison of the low-spin components of various NO-ferrohemes. **a** ​​Simulated EPR spectrum. Red: Experimental EPR data of alb-heme-NO (Figure 1b, bottom). Blue: Simulated 5-C heme(Fe^2+^)-NO ({FeNO}^7^) spectrum (g = 2.074, 2.030, 2.008; _s_g = 0.0115, 0.0080, 0.0004; A = 20, 20, 51 MHz). Green: Simulated GS-heme-OH_2_ spectrum (g = 2.280, 2.158, 1.935; _s_g = 0.042, 0.018, 0.023). Simulation was performed using SpinCount, a software developed by the Hendrich group at Carnegie Mellon University. **b** EPR spectrum of alb-heme-NO without purification of G-25 column. **c** EPR spectrum of alb-heme-NO purified with G-25 column. **d** EPR spectrum of HbNO purified with G-25 column. **e** EPR spectrum of MbNO purified with G-25 column. HbNO was a mixture of 5-C and 6-C, while MbNO was 6-C.


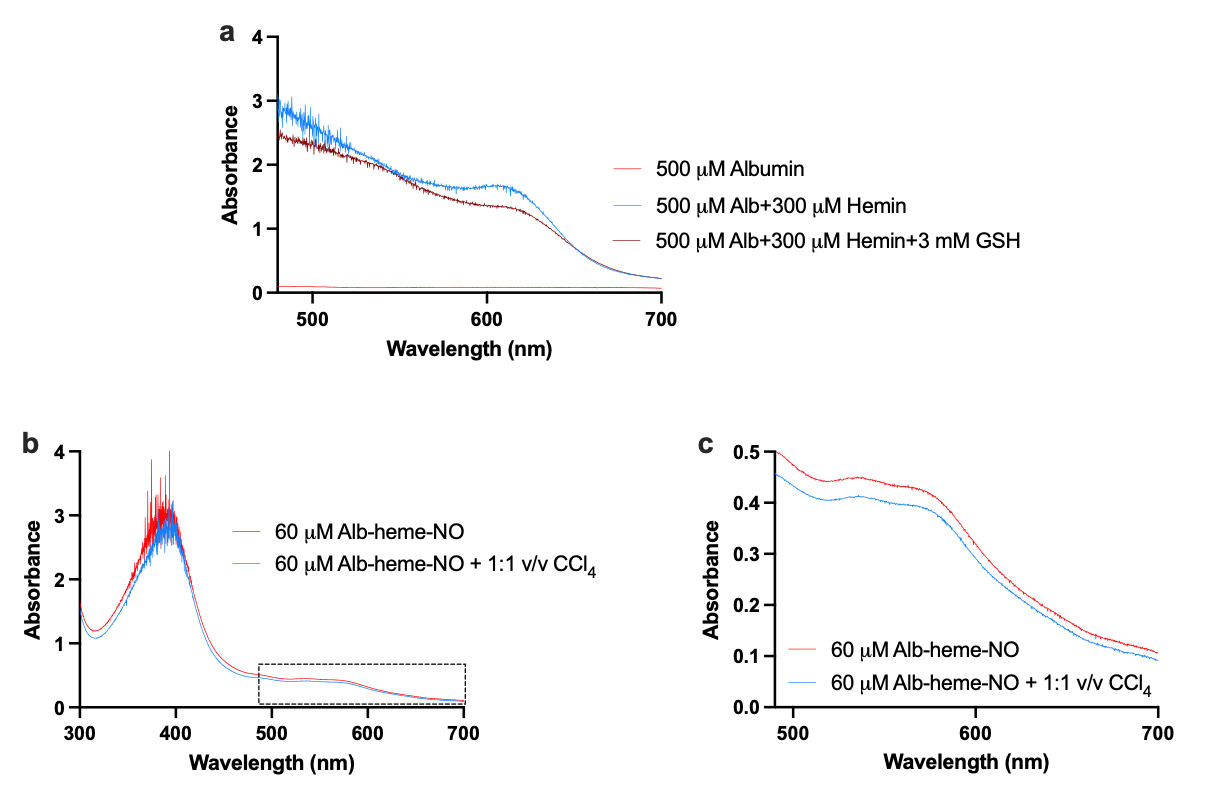


Figure S2. Representative UV-Vis spectra of the reaction mixtures. **a** Spectral changes of 300 µM hemin upon addition of 3 mM GSH. **b** Spectra of 60 µM alb-heme-NO before and after protein precipitation with CCl_4_. **c** Enlarged view of the region indicated by the dashed rectangle in panel **b**, highlighting spectral features of interest. Panels **b–c** demonstrate that CCl_4_-induced precipitation removes albumin, while alb-heme-NO remains in the aqueous phase, showing no detectable change in its spectral properties compared to the pre-precipitation sample. All UV‒visible spectroscopic scans were performed on a Varian Cary 50 (Agilent Technologies, Inc., Santa Clara, CA).


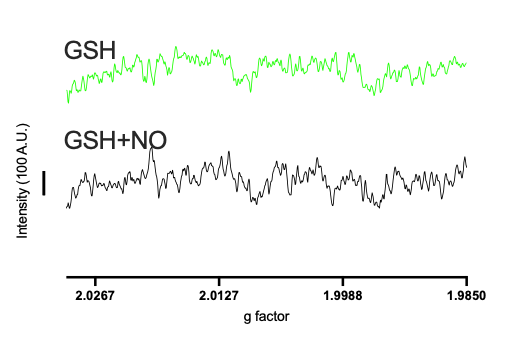


Figure S3. EPR spectra of **a** GSH (3mM) and **b** GSH in reaction with NO (600 μM). NO facilitated generation of thiyl radical from GSH.


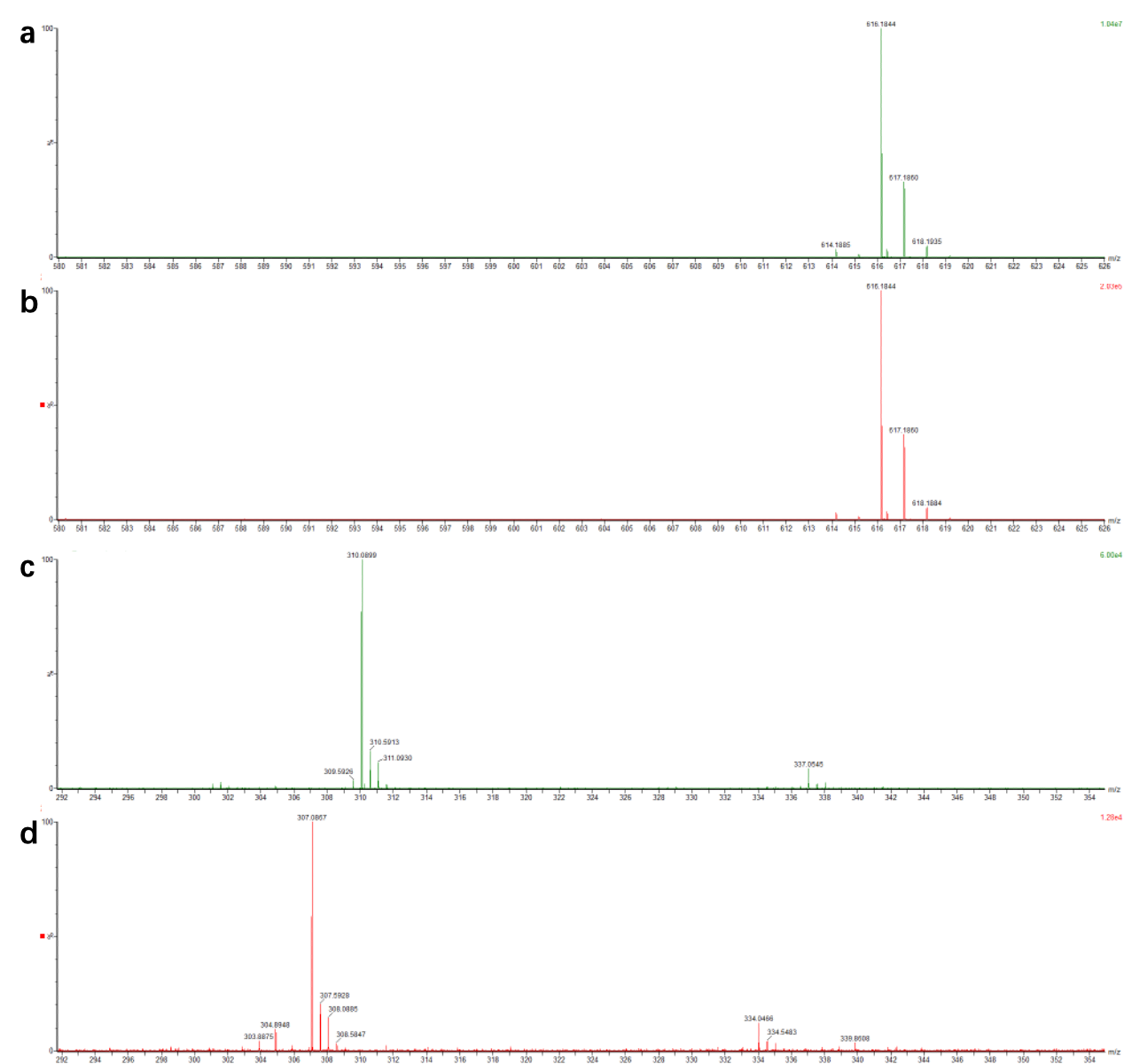


Figure S4. MS detection of heme (**a-b**) and GSH (**c-d**) in the small molecules-removed and albumin-precipitated heme-NO. **a** and **c** are results of alb-heme-NO synthesized with isotopically labeled (^13^C_2_, ^15^N_1_) GSH, while **b** and **d** are results of alb-heme-NO synthesized with unlabeled GSH.


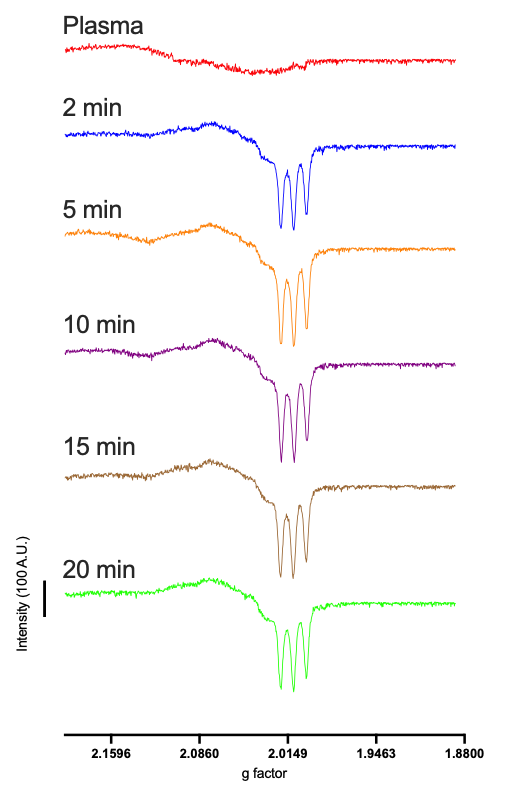


Figure S5. Representative EPR spectra of plasma at different time points after addition of 20 μM alb-heme-NO.


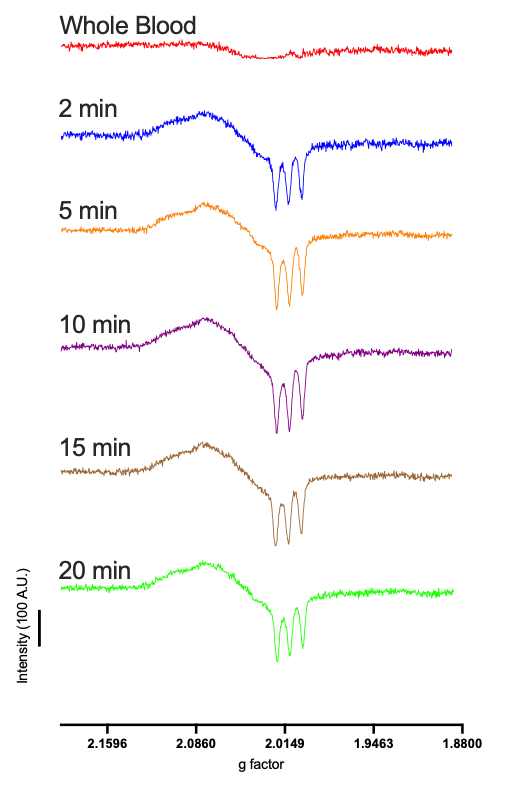


Figure S6. Representative EPR spectra of whole blood at different time points after addition of 20 μM alb-heme-NO.


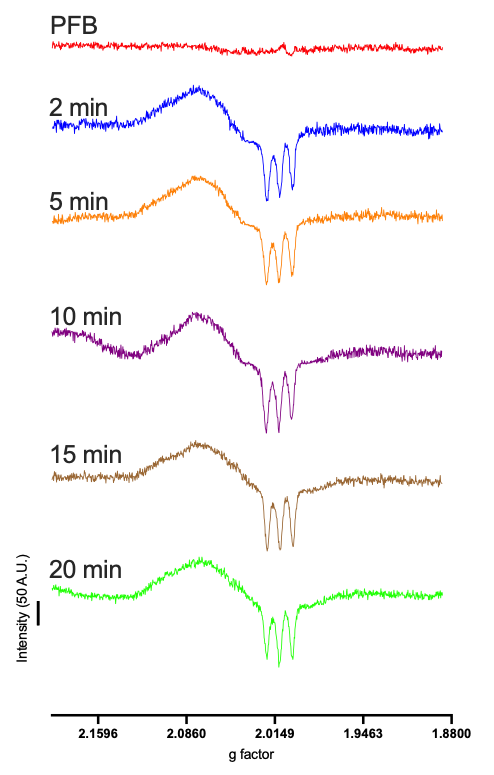


Figure S7. Representative EPR spectra of plasma-free blood (PFB) at different time points after addition of 20 μM alb-heme-NO. Although not specifically investigated, the rhombic signals observed at 10 min may result from RBC lysis during the sample freezing process, a necessary step in EPR sample preparation.


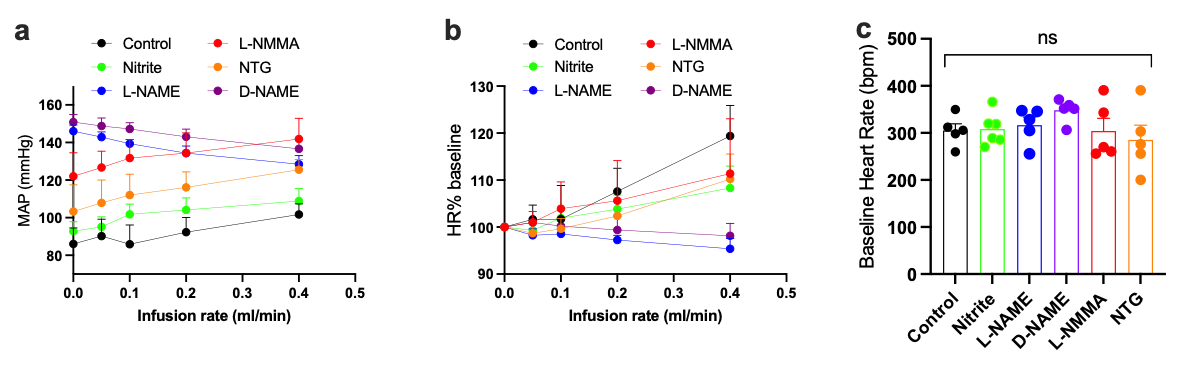


Figure S8. Effects of alb-heme-NO stepwise infusion on mean arterial blood pressure (MAP; **a**) and heart rate (HR; **b** for relative change, **c** for absolute value at baseline).


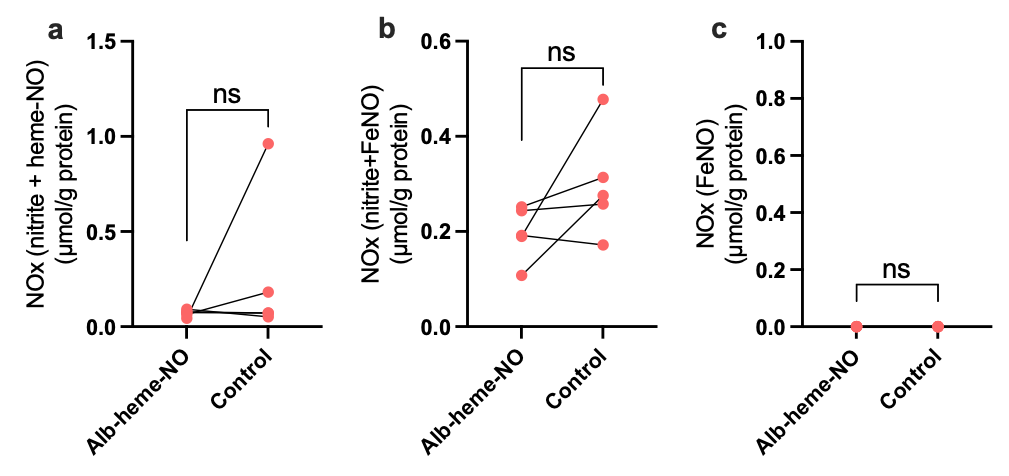


Figure S9. Chemiluminescence measurements of different NOx in arterial homogenates. **a** VitC+HAc methodology. **b** FeCN+HAc methodology. **c** FeCN+Hepes methodology.


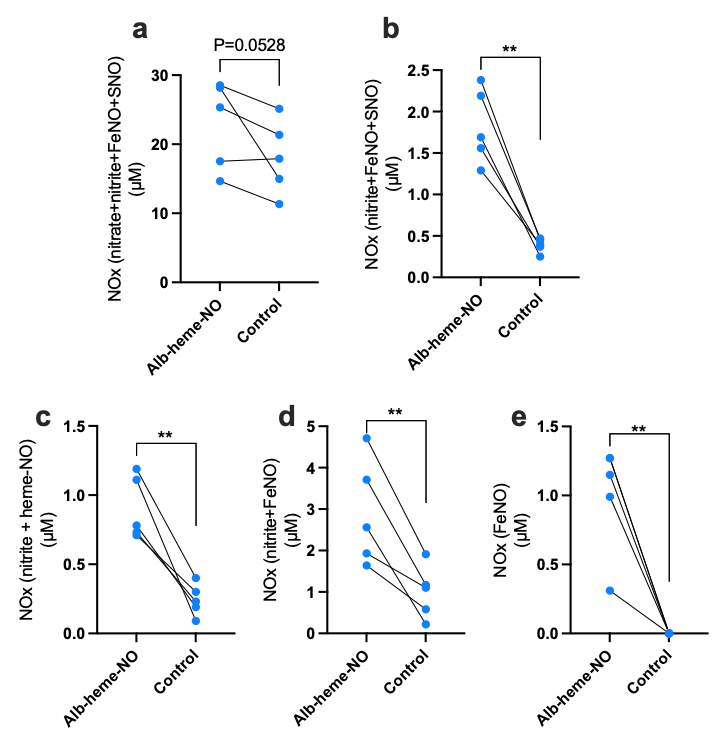


Figure S10. Chemiluminescence measurements of different NOx in the luminal buffer at the end experiments. **a** NiR+I_3_^-^ methodology. **b** I_3_^-^ methodology. **c** VitC+HAc methodology. **d** FeCN+HAc methodology. **e** FeCN+Hepes methodology. ** =p *<*0.01, paired t-test.


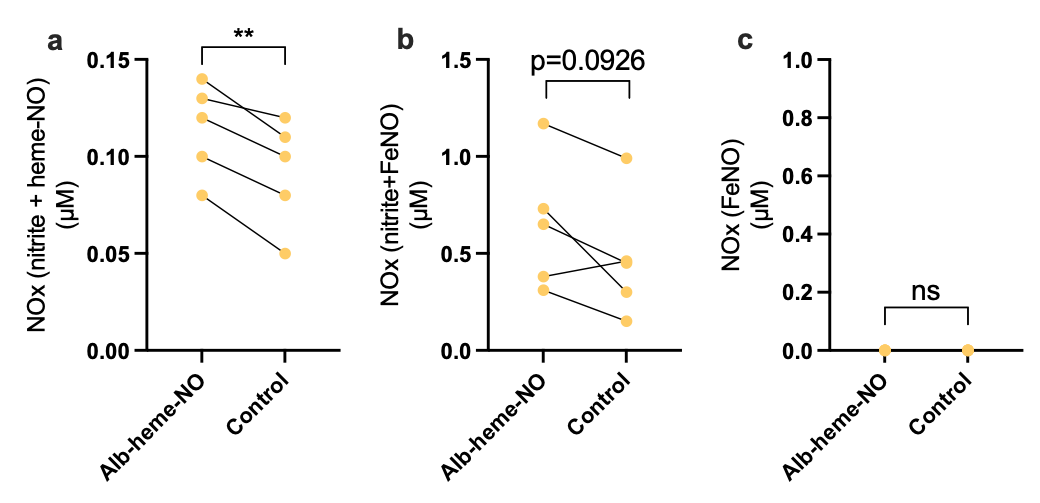


Figure S11. Chemiluminescence measurements of different NOx in the abluminal buffer at the end experiments. **a** VitC+HAc methodology. **b** FeCN+HAc methodology. **c** FeCN+Hepes methodology. ** =p *<*0.01, paired t-test.


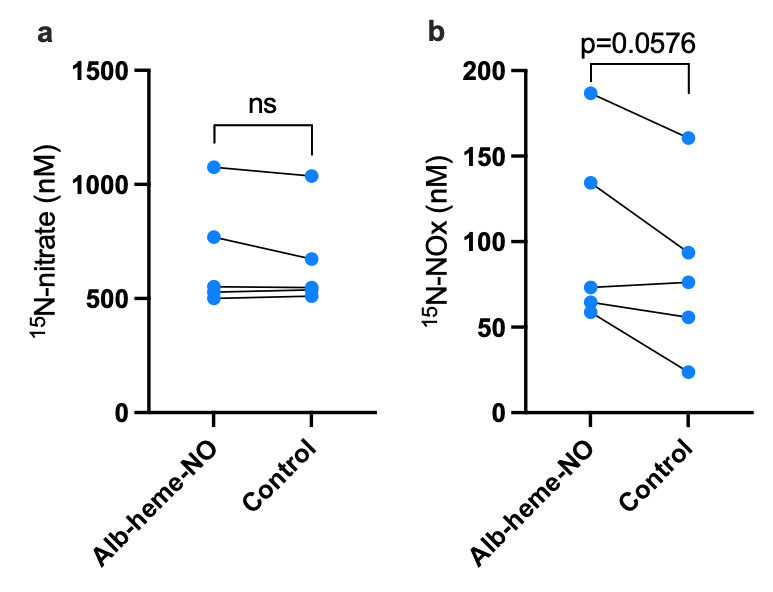


Figure S12. GC-MS measurements of different NOx in the luminal buffer at the end experiments. **a** ^15^N-nitrate. **b** ^15^N-NOx including nitrite, SNOs, and FeNO.


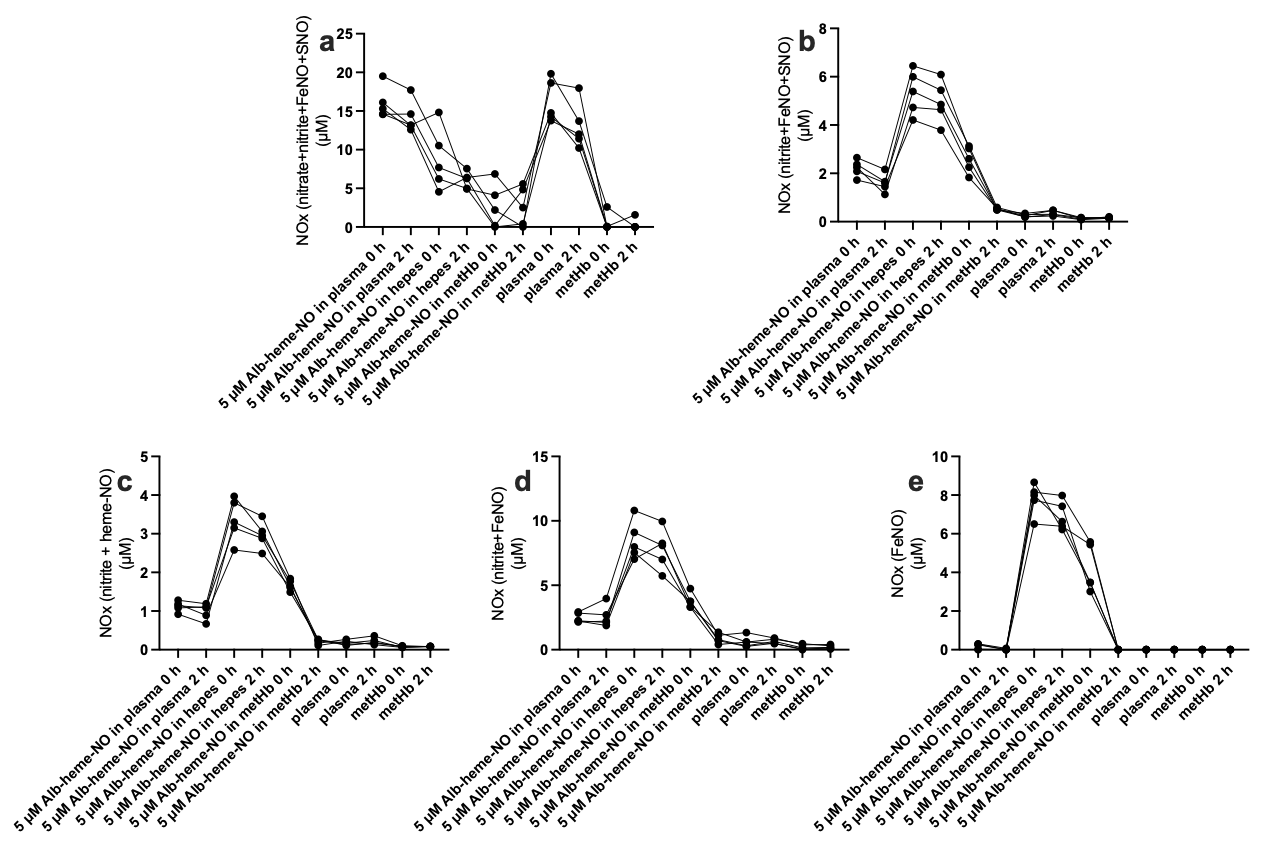


Figure S13. Chemiluminescence measurements of different NOx in the various buffers used in experiments. **a** NiR+I_3_^-^ methodology. **b** I_3_^-^ methodology. **c** VitC+HAc methodology. **d** FeCN+HAc methodology. **e** FeCN+Hepes methodology. 0 h represents samples collected before experiments, while 2 h represents those collected at the end of experiments.

**References:**

1. [Liu T, Zhang M, Terry MH, Schroeder H, Wilson SM, Power GG, Li Q, Tipple TE, Borchardt D, Blood AB. Nitrite potentiates the vasodilatory signaling of S-nitrosothiols. *Nitric Oxide* 2018;**75**:60–69.](http://paperpile.com/b/irc4iT/9H5L)

2. [Liu T, Zhang M, Terry MH, Schroeder H, Wilson SM, Power GG, Li Q, Tipple TE, Borchardt D, Blood AB. Hemodynamic Effects of Glutathione-Liganded Binuclear Dinitrosyl Iron Complex: Evidence for Nitroxyl Generation and Modulation by Plasma Albumin. *Mol Pharmacol* 2018;**93**:427–437.](http://paperpile.com/b/irc4iT/4AITK)

3. [Liu T, Zhang M, Duot A, Mukosera G, Schroeder H, Power GG, Blood AB. Artifacts Introduced by Sample Handling in Chemiluminescence Assays of Nitric Oxide Metabolites. *Antioxidants (Basel)* 2023;**12**.](http://paperpile.com/b/irc4iT/Ku32c)

4. [Mukosera GT, Liu T, Ishtiaq Ahmed AS, Li Q, Sheng MH-C, Tipple TE, Baylink DJ, Power GG, Blood AB. Detection of dinitrosyl iron complexes by ozone-based chemiluminescence. *Nitric Oxide* 2018;**79**:57–67.](http://paperpile.com/b/irc4iT/RlPUx)

5. [Liu T, Zhang M, Mukosera GT, Borchardt D, Li Q, Tipple TE, Ishtiaq Ahmed AS, Power GG, Blood AB. L-NAME releases nitric oxide and potentiates subsequent nitroglycerin-mediated vasodilation. *Redox Biol* 2019;**26**:101238.](http://paperpile.com/b/irc4iT/pXlkc)

6. [Liu T, Zhang M, Li Q, Schroeder H, Power GG, Blood AB. Nitrite reverses nitroglycerin tolerance via repletion of a nitrodilator-activated nitric oxide store in vascular smooth muscle cells. *Redox Biol* 2025;**80**:103513.](http://paperpile.com/b/irc4iT/FWR7v)
